# Extra gastrointestinal stromal tumor EGIST in the recto-vesical pouch: A case report and literature review

**DOI:** 10.1016/j.amsu.2022.103283

**Published:** 2022-01-25

**Authors:** Abdelhakim Harouachi, Marouane Harhar, Mohammed Mhand, Abderrahman Atmani, Anass Elamrani, Ayoub Kharkhach, Tariq Bouhout, Badr Serji, Tijani EL. Harroudi

**Affiliations:** a Surgical Oncology Department, Regional Oncology Center, Mohammed VI University Hospital, Oujda, Morocco; b Mohammed First University Oujda, Faculty of Medicine and Pharmacy Oujda, Oujda, Morocco

**Keywords:** GIST, Gastrointestinal stromal tumors, EGIST, Extra gastrointestinal stromal tumors, GIST, EGIST, Abdomen and pelvic mass, Recto-vesical pouch, Immunohischimical study, Resection

## Abstract

**Introduction:**

Extra-gastrointestinal stromal tumors (EGISTs) are rare mesenchymal tumors located outside the gastrointestinal tract, and exhibit the same phenotypic and morphological profile of gastrointestinal stromal tumors (GISTs).

**Case report:**

We report the case of a 20-year-old male patient consulted for chronic discomfort in the hypogastric region. Abdominal ultrasound and abdomino-pelvic CT scan identified a retro-vesical mass measuring 16 × 9 cm. He underwent an exploratory laparotomy and a total resection of the mass R0. The histopathological panel of the surgical specimen confirmed the diagnosis of EGIST.

**Clinical discussion:**

The primary localization in the recto-vesical pouch of EGIST is a very rare entity. Their clinical and radiological presentations are unusual, and their definitive diagnosis is largely based on immunohistochemistry staining.

**Conclusion:**

the origin of extra gastrointestinal stromal tumors EGIST can remain unclear.

## Highlights

• Extra gastrointestinal stromal tumors (EGISTs) are very rare primary entities that develop outside the digestive tract.
• Their primary localization in the recto-vesical pouch is a very rare entity.
• Their definitive diagnosis is largely based on immunohistochemistry staining.
• The only accepted treatment for non-metastatic EGISTs is a complete surgical resection.

## 1 Introduction

Gastrointestinal stromal tumors (GISTs) are rare connective tissue tumors resulting from the anarchic proliferation of Cajal interstitial cells [1]. They can affect all segments of the gastrointestinal tract, and constitute less than 1% of tumors in the digestive tract. Extra gastrointestinal stromal tumors (EGISTs), similar to the morphological and phenotypic characters of GISTs, are very rare primary entities that develop outside the digestive tract. They represent less than 5% of stromal tumors of GI tract [2]. Over 80% of EGISTs are located in omental bursa and mesentery [3].

The diagnosis is often confirmed only after the immunohistochemical study. A complete surgical resection with negative margin R0 is the gold standard treatment of GISTs and EGISTs [2]. Here, we report an interesting case of an EGIST arising in the retro-vesical pouch, as an unusual localization, in a 20-year-old male patient. It was revealed by a large abdomino-pelvic mass, and exercises a mass effect on the small bowel. Based on a literature review, we discuss the diagnosis, pathogenesis and treatment of this condition. This case has been reported following the SCARE criteria [4].

## 2 Case presentation

A 20-year-old male patient, with no particular pathological history, was referred to the surgical oncology department for the management of an abdomino-pelvic mass. The patient was reported a chronic discomfort in the hypogastric region, and later low back pain developed for a duration of four months. It was accentuated at the time of defection, initially neglected, without any other digestive symptom or any symptom of gastrointestinal obstruction or urinary disorder. Notably absent were symptoms of vomiting, fever, or weight loss. There are no proven genetic abnormalities in his family, and the history of cancer was unremarkable. He had no history of drug use, tobacco, or alcohol abuse.

During the clinical examination, the patient was hemodynamically and respiratory stable, apyretic. Her body mass index (BMI) was 23.1 kg/m^2^. Abdominal examination revealed an abdominal distension with tenderness of the hypogastrium, without collateral venous circulation. There was a large, firm, non-pulsatile, tender palpable abdominal mass behind the hypogastrium and the umbilicus. The lymph node areas were free. The findings of laboratory tests, including Carcinoembryonic Antigen, Carbohydrate Antigen 19.9 (CA 19.9) Carcinoma Antigen 125 (CA 125), were unremarkable.

**Fig. 1:**
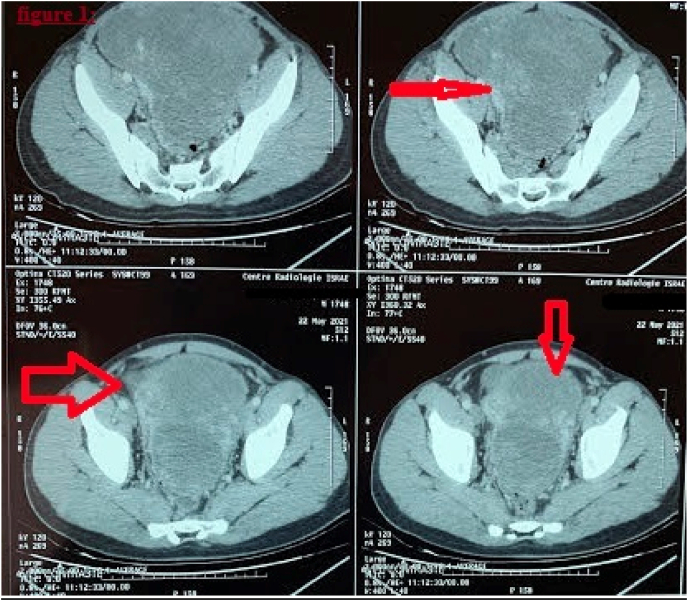
Enhanced Abdomino-pelvic computed tomography demonstrates a voluminous mass centered on the retro-bladder region (arrow).

Abdominal ultrasound showed a voluminous mass, heterogeneous, coarsely rounded, and hyper-vascularised on Doppler with millimeter cystic formations. An abdomino-pelvic computed tomography CT was performed, objecting a voluminous mass centered on the retro-bladder region (Fig. 1). This lesion was a well-defined, heterogeneous with largely cystic necrotic areas and hypo-dense areas enhanced after injection of contrast product. It has pushed the bladder forward, in intimate contact with the rectum, pushed back the small bowel without any sign of noticeable invasion. It measures 16 × 9 cm axially, extended in height over 18.7cm (Fig. 1).

A first ultrasound-guided biopsy with Tru-cut® was performed, but this one was non-contributory, and the second showed, after pathological examination, a benign angiomatous tissue.

**Fig. 2:**
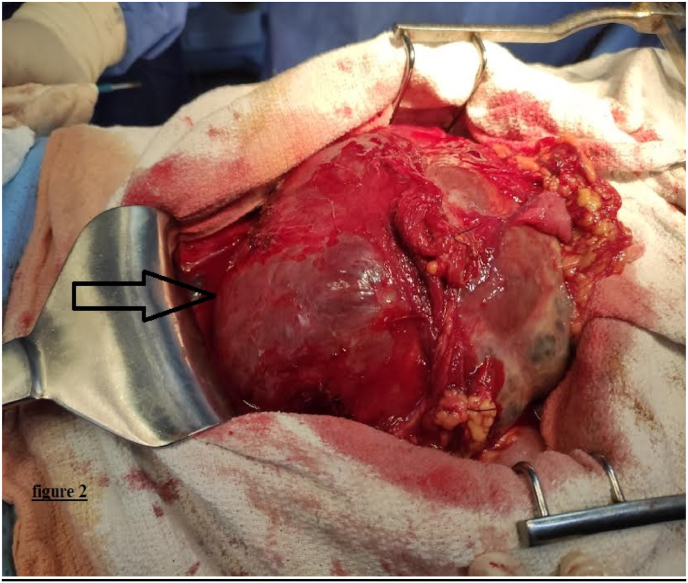
Intra-operative view of a solido-cystic tumor that was found in the recto vesical pouch (arrow).

An exploratory laparotomy was finally indicated in view of this unspecified histological aspect. On exploration, an enormous retro-bladder mass measuring approximately 18 cm in all its dimensions, solido-cystic, of necrotico-brownish appearance with foci of hemorrhagic reshaping. The tumor was very adherent in the anterior to the bladder, compressing the small bowel and omentum upwards, in contact with the rectum behind (Fig. 2). This mass was dissected from the small bowel, the bladder, and the rectum after section ligation of the nutrient vessels, and aspiration of intracystic fluid. The tumor was completely resected from the recto-vesical pouch, and a suction drainage tube was placed in this pouch. Total operative time was 115 min, with an estimated blood loss of 30 ml. The procedure was performed by a professor of surgical oncology with over 25 years of surgical experience at an academic hospital.

The postoperative follow-up was without incident and the patient was discharged from the hospital on day 11.

**Fig. 3:**
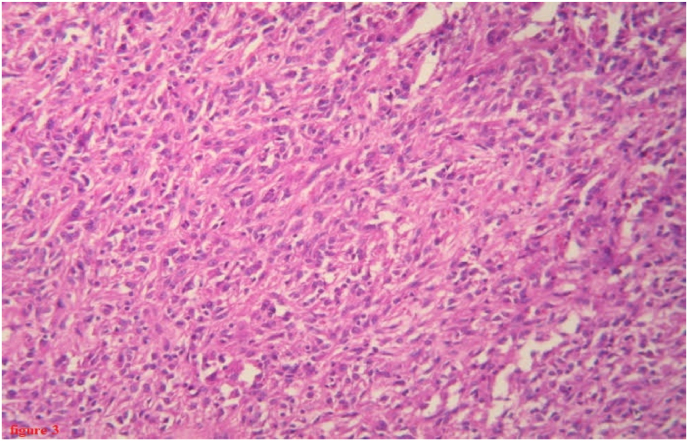
Photomicrograph (HES × 20) showing proliferation of spindle-shaped cells.

Macroscopically, the mass weighed 540g with double capsule. On the cut surface, it revealed an irregular, soft tumor with thick-walled and hemorrhagic areas. On microscopic sectional examination, the tumor proliferation was largely dissociated by cystic and hemorrhagic changes with heterogeneous density, composed of oblong cells, most often spindle-shaped with eosinophilic cytoplasm. The nuclei of these cells are not atypical with a low mitotic activity estimated at 4 mitoses per 50 fields at magnification × 40. These cells organize themselves into bundles of varying sizes in an angiomatous stroma with numerous congestive and hemorrhagic rearrangements (Fig. 3).

**Fig. 4:**
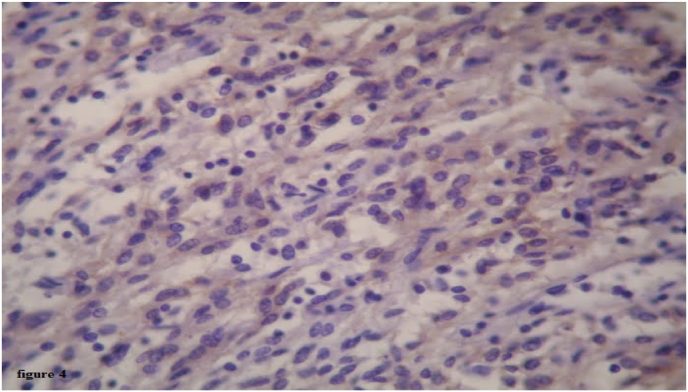
Immunohistochemical analysis showed diffuse strong CD117 positivity for both cytoplasmic and membranous components.

The immunohistochemistry staining was positive for the anti-DOG1 antibodies, the anti-CD34 antibodies, the anti-CD117 antibodies, and negative for anti-CD31 antibodies, and labeling estimated at approximately 3% of tumor cells for *anti*-Ki67 antibodies (Fig. 4).

These pathological and immunohistochemical aspects suggested the diagnosis of an EGIST.

The patient was started adjuvant therapy with Imatinib 400 mg, and was seen again in consultation after 6 months. The clinical and radiological examination is unremarkable. The outcome was favorable without tumor recurrence.

### 2.1 Clinical discussion

Primary extra-gastrointestinal stromal tumors (EGISTs) are extremely rare mesenchymal tumors resulting from any location outside the gastrointestinal tract, and exhibit the similar histological and immunohistological behaviors and principles of gastrointestinal stromal tumors (GISTs). Overall, these entities of recent description are rarely primitive, their etiology is still poorly understood, and can be found in the retro-peritoneum, the abdominal wall, the mesentery, and the prostate [5,6]. To the best of our knowledge, few cases of EGIST of undetermined origin were reported in the literature. We report the first case from Morocco of EGIST in the recto-vesical pouch with an undetermined origin treated by R0 resection after dissecting of the mass from all adjacent tissues.

EGISTs are developed from ICC-likes cells that would express a tyrosine kinase receptor called CD117 (KIT protein) almost constantly. The "gain-of-function" mutation involves 80–86% in oncogenesis of GISTs by affecting the c-Kit gene [7,8].

The clinical symptomatology of EGIST is widely variable, depending on the primary location, loco-regional extent, and tumor size. Patients with EGIST can remain asymptomatic for a long time. The clinical signs are not very specific. Hypogastric discomfort with pelvic pain can manifest as in our case. The main revealing symptoms are abdominal pain, the discovery of an abdominal mass, gastrointestinal bleeding, and more rarely an acute abdominal picture or occlusive syndrome [9]. Radiological exploration by computed tomography is the reference examination for visualizing the tumor and its reports and deciding the treatment protocol. The exact origin of EGISTs is not always clear and precise [10,11]. In our case, the origin was unclear in the pelvic area.

Pathological examination of stromal tumors shows 3 forms: epithelioid form, spindle form, and mixed form [12]. In contrast to GIST, the epithelioid contingent is more present in EGIST. The immunohistochemical study is a valuable tool for validating the diagnosis based on positive immunostaining for c-KIT (CD117) in 90–95% of cases [13].

The best and only accepted treatment for non-metastatic EGISTs is a complete surgical resection. The laparoscopic approach is inappropriate due to the lack of courageous results. Taking into account prognostic factors, macroscopically complete resection R0 with healthy margins is required [2]. In our case, imatinib was prescribed because of the high risk of recurrence of stromal tumors.

For locally advanced or metastatic EGISTs, the therapeutic protocol comprising imatinib as a neo-adjuvant is necessary for tumor reduction. A complete or partial response to imatinib according to the criteria of RECIST is an indication for resection as adjuvant therapy [14,15].

Peritoneal dissemination occurs following tumor invasion. It is the main risk of resection of the mass, and modifies the prognosis and overall survival [2].

According to Miettinen's classification, the location of GISTs is a significant prognostic factor. The EGIST has a worse prognosis. Tumors with a size greater than 5 cm in its large diameter have usually a worse prognosis. The behavior of EGISTs is more aggressive when the mitotic index estimated at 5 by 50 fields at magnification and a Ki67 index greater than 10% [16].

## 3 Conclusion

The origin of extra gastrointestinal stromal tumors EGIST can remain unclear. This case illustrates the need to multi-center collaborative studies for the management of these entities.

## Ethical approval

No ethical approval necessary.

## Sources of funding

The author(s) received no financial support for the research, authorship and/or publication of this article.

## Author contribution

All authors contributed toward data analysis, drafting and revising the paper, gave final approval of the version to be published and agree to be accountable for all aspects of the work.

## Author statement

Dr Harouachi Abdelhakim: Have written the article, have consulted the patient, prescribed all of the tests and prepared the patient for surgery and participated in the surgery.

Dr Marouane Harhar: have helped writing the article, data collection. Dr Mhand Mohammed: have helped writing the article, data collection.

Dr Atmani Abderrahman: have helped writing the article, data collection. Dr Elamrani Anass: have helped writing the article, data collection.

Dr Kharkhach Ayoub: have helped writing the article, data collection.

Pr Bouhout Tariq (oncology surgery professor): supervised the writing of manuscript. Pr Serji Badr (oncology surgery professor): supervised the writing of manuscript.

Pr El Harroudi Tijani (oncology surgery professor): have supervised the writing of the paper, and had been the leader surgeon of the case.

## Registration of research studies

Our paper is a case report; no registration was done for it.

## Patient consent

Written informed consent was obtained from the patient for publication of this case report and accompanying images. A copy of the written consent is available for review by the Editor-in-Chief of this journal on request.

## Provenance and peer-review

Not commissioned, externally peer reviewed.

## Consent

We have requested the patient's consent to publish this case report for educational purposes.

## Guarantor

Abdelhakim Harouachi.

## Declaration of competing interest

The authors declare that they have no conflict of interest.
